# Effect of a community-based lifestyle intervention on predictors of behavior change regarding a healthy plant-based diet—The Healthy Lifestyle Community Program (cohort 2)

**DOI:** 10.3389/fpubh.2025.1560376

**Published:** 2025-10-06

**Authors:** Carmen Kettler, Ragna-Marie Weber, Corinna Anand, Sarah Husain, Christian Koeder, Nora Schoch, Maren M. Michaelsen, Tobias Esch, Heike Englert

**Affiliations:** ^1^Department of Nutrition Sciences, University of Applied Sciences Münster, Münster, Germany; ^2^Institute for Integrative Health Care and Health Promotion (IGVF), Faculty of Health, School of Medicine, Witten/Herdecke University, Witten, Germany; ^3^Institute for Prevention and Cancer Epidemiology (IPE), Faculty of Medicine and Medical Center, University of Freiburg, Freiburg im Breisgau, Germany

**Keywords:** community-based lifestyle intervention, plant-based diet, physical activity, stress management, self-efficacy, non-communicable diseases (NCDs)

## Abstract

**Background:**

Implementing healthy behaviors, particularly a healthy plant-based diet, can play a key role in preventing non-communicable diseases (NCDs). However, behavior change initiation and maintenance can be challenging. The objective was to test, if the Healthy Lifestyle Community Program (cohort 2; HLCP-2) was effective in changing psychological constructs regarding eating behavior.

**Methods:**

A 24-month non-randomized controlled intervention study with a community-based approach in rural Germany was conducted. The intervention group (IG) received a 10-week intensive lifestyle intervention aiming to improve NCD risk factors, followed by a 22-month alumni phase. The control group (CG) received no intervention. Participants completed questionnaires at six measurement time points to assess psychosocial constructs of behavior change derived from the Health Action Process Approach, including action/coping planning, action self-efficacy (SE), maintenance SE, recovery SE, and eating behavior. An exploratory analysis with inter- and intra-group comparisons regarding different scores of HAPA constructs was conducted. Covariate-adjusted comparisons were performed using multiple linear regression models. Additionally, bivariate correlations between these constructs and the healthy plant-based diet index (hPDI) were examined.

**Results:**

A total of 186 participants (IG: *n* = 111; CG: *n* = 75) were analyzed. In the IG, all HAPA scores increased significantly at all measurement time points compared to baseline, with the highest impact after the intensive phase (*p* < 0.001). Between-group comparisons for action/coping planning and action SE were significant at all measurement time points, while results for maintenance and recovery SE were inconsistent in the study course. Adjusting for covariates did not substantially alter the results. After 10 weeks, only recovery SE correlated significantly with hPDI (ρ = 0.289, *p* = 0.004).

**Conclusion:**

Participation in the HLCP-2 intervention resulted in improvements in planning health-promoting behaviors, action, maintenance, and recovery SE. Further research is required to determine whether an increase in action planning and SE leads to changes in dietary behavior.

**Clinical trial registration:**

German Clinical Trials Register DRKS (https://drks.de/search/de; reference: DRKS00018775; retrospectively registered).

## Background

Non-communicable diseases (NCDs) such as type 2 diabetes mellitus, cardiovascular diseases, or cancer are considered the greatest challenge facing global health care systems in the twenty-first century, accounting for approximately 41 million deaths per year worldwide (1). Despite intensified efforts to prevent and treat these diet- and lifestyle-dependent diseases, their prevalence is increasing (2). It is well established that the individual lifestyle has a great impact on the development and course of NCDs and their risk factors, such as obesity, hypertension, dyslipidemia, and impaired glucose tolerance (3, 4). A shift toward healthy behaviors, i.e., a healthy plant-based diet, sufficient physical activity, smoking cessation, stress management, and body weight regulation, can thereby maintain health as well as help slow or even reverse the development of chronic diseases (5–9). Therefore, such lifestyle behavior changes are the target of numerous prevention and health promotion interventions (1, 10).

However, interventions designed to change health-related behaviors often yield modest effects and show inconsistent results, especially regarding long-term effects (11, 12). Designing effective interventions to change health behaviors requires a strong integration of theoretical models and a deeper understanding of the underlying mechanisms of behavior change. A challenge lies in understanding the functional mechanisms of different behavior change techniques (BCTs) and how they address resources, e.g., self-efficacy (SE), and therefore change actual health behavior (12, 13).

Many theoretical models have been developed to explain the mechanisms underlying health behavior change. One such model is the Health Action Process Approach (HAPA), which outlines specific predictors and mechanisms for initiating and maintaining health behavior change (14). The HAPA model is particularly useful because it distinguishes between two distinct but interconnected phases: the motivation phase, where individuals form an intention to change their behavior, and the volition phase, where they translate this intention into action. Both intention forming and action are influenced by several psychological constructs. Key predictors in the motivation phase include outcome expectancy, which represents the anticipated consequences of a behavior (positive or negative), and action SE, defined as the belief in one's ability to perform the required behavior successfully (14). The motivation phase concludes with an intention for behavior change. Although a behavioral intention is a relevant predictor of behavior, it does not guarantee action. Many individuals struggle to move beyond their intentions, a phenomenon called the intention-behavior gap (12, 14). Once an intention has been formed, individuals enter the volition phase, where the HAPA model focuses on the processes that help translate intentions into action and sustain those action over time. Within this phase, action and coping planning play key roles. Action planning involves creating specific plans about the performance of a behavior, while coping planning focuses on obstacles and devising strategies to overcome them. In addition, maintaining behavior over the long term depends on maintenance SE, the confidence to persist in a behavior despite challenges, and recovery SE, the belief in one's ability to resume the behavior after setbacks (15). The HAPA constructs relevant for this study, along with their definitions and exemplary items, are presented in Table 1. A comprehensive visual representation of the HAPA model can be found in Schwarzer (14).

**Table 1:** Explanation and description of HAPA constructs used in the analysis (15, 48) (HAPA, Health Action Process Approach; SE, self-efficacy).

**HAPA constructs**	**Explanation**	**Exemplary item**	**Number of items**	**Min. and max. score**	**Cronbach's alpha**
Outcome Expectancy	Understanding of the subsequent outcome of a specific behavior	What are the (dis-) advantages of eating healthy for you personally? e.g., If I eat healthy, it is good for my blood parameters (e.g., blood sugar or cholesterol levels)	Descriptive results	Not applicable	Not applicable
Action SE	Optimistic belief of a person to be successful (pre-action)	How confident are you in your ability to consume fruits, vegetables, and whole grains on a regular basis?	4	4–16	*t*_0_: 0.723; *t*_1_: 0.785; *t*_2_: 0.825; *t*_3_: 0.824; *t*_4_: 0.800; *t*_5_: 0.828
Action/coping planning	Envisioning of a detailed plan (action planning) and developing explicit strategies for accomplishing the task even with obstacles (coping planning)	Please think about a healthy diet. Have you (already) made concrete plans about which foods you should prefer to eat?	6	6–24	*t*_0_: 0.827; *t*_1_: 0.865; *t*_2_: 0.892; *t*_3_: 0.871; *t*_4_: 0.897; *t*_5_: 0.896
Maintenance SE	Handling of hurdles that emerge during the phase of maintaining a new behavior	How confident are you that you can eat healthfully in the long run, even if you eat away from home (e.g., at a friends' house, at a party, at a restaurant)?	12	12–48	*t*_0_: 0.877; *t*_1_: 0.928; *t*_2_: 0.935; *t*_3_: 0.925; *t*_4_: 0.944; *t*_5_: 0.948
Recovery SE	Optimistic belief to deal with failure and recover from setbacks and the confidence to restart the learned behavior	What about once you have “sinned”? How confident are you that you can eat healthily again, even if you once ate unhealthily for several days?	3	3–12	*t*_0_: 0.904; *t*_1_: 0.891; *t*_2_: 0.942; *t*_3_: 0.942; *t*_4_: 0.961; *t*_5_: 0.961

SE items scored on a four-point Likert scale from “not confident (1)” to “completely confident (4)”. Action/coping planning items scored on a four-point Likert scale from “not at all (1)” to “firmly (4)”.

Maintaining behavior over the long term is regarded as one of the most challenging aspects of behavior change. The time span at which behavior is considered “maintained” varies across theories and studies. However, 6 months of sustained behavior is often used as a key threshold for successful maintenance, as seen in models like the Transtheoretical Model (16, 17). The HAPA model itself does not explicitly specify a time frame for behavioral maintenance (18). Many studies on interventions targeting healthy eating behaviors predominantly focus on short-term effects during or immediately following the intervention, with follow-ups typically conducted at around 3 months or, at most, 6 months [e.g., (19, 20)]. Long-term assessments beyond 1 year remain scarce, leaving a gap in understanding the process of maintaining healthy eating behaviors.

To address this, we conducted a 24-month controlled intervention study: The Healthy Lifestyle Community Program (cohort 2; HLCP-2), an intensive lifestyle intervention with a community-based approach. The first cohort of the HLCP (HLCP-1; 2017–2019) served as the initial implementation of the program and featured a slightly different version of the lifestyle intervention compared to HLCP−2. Findings from HLCP-1 informed refinements made to the intervention design in HLCP-2 and have been previously published (21, 22). The intervention aimed to reduce body weight and improve NCD risk profile by improving individual health behaviors of participants. The primary outcome of the study, change in body weight, has been previously published (23, 24). A significant reduction in body weight was observed both after 10 weeks and 1 year compared to baseline and the control group (CG). The aim of this analysis is to investigate the long-term effect (2 years) of the HLCP-2 intervention on psychological constructs regarding dietary habits derived from the HAPA model and the impact of these constructs on eating behavior, assessed using a semi-quantitative 3-day food record. The aligned hypotheses state a significant change of action planning as well as action, maintenance, and recovery SE in the intervention group compared to baseline and control at all measurement time points (10 weeks, 6, 12, 18, and 24 months). Although the intervention is expected to produce effects in one direction (improvement of HAPA scores), two-sided tests were chosen to allow for the detection of unexpected or context-specific effects. In addition, we hypothesize a positive correlation between changes in HAPA constructs and the healthy plant-based diet index (hPDI).

## Methods

### Study design

The study was designed as a non-randomized, controlled study with a total duration of 24 months (2018–2020). The study was conducted in two municipalities in north-west Germany: one “intervention community” and one “control community” to ensure that the participants in the CG were unaware of the intervention. As part of the community-based approach, a health circle was established in the intervention community prior to the implementation of the intervention. This health circle brought together local health stakeholders, including representatives from healthcare, community organizations, and local government, to discuss relevant health topics in order to develop the content of the intervention. The mayor of the community was included, which was instrumental in representing community interests. However, due to the complexity of the real-world approach, which involved local stakeholders during the planning phase and prior of recruitment, cluster randomization was not feasible [as described previously: (25)]. As is common in lifestyle interventions, blinding of participants and/or instructors was not possible (26).

### Participants

Recruitment took place at a health market, a community event that provided an interactive platform to raise awareness of health initiatives in the community and engage potential participants. We also used posters and newspaper articles in the intervention community. In the control municipality, the recruitment took place at a local event. Inclusion criteria were merely adult age (≥18 years) and physical and mental ability to participate in the study (27). Due to the real-world approach of the study, we included not only participants who were overweight, obese, or at high risk for NCDs, but also individuals with normal weight. Participants were allocated to the IG and CG based on their place of residence (intervention vs. control community).

### Intervention

The HLCP-2 intervention consisted of a 10-week intensive intervention and a 22-month follow-up phase primarily addressing salutogenesis and individual health resources. The theoretical basis of the intervention was the Health Action Process Approach (15). The intervention followed four guiding principles for health behavior: a healthy, plant-based diet (8), physical activity (28), stress management (9), and community support (27).

Dietary recommendations of the HLCP-2 intervention were diets high in vegetables and fruits, whole grains, legumes, nuts, seeds, healthy oils, and low in meat, high-fat dairy, highly processed foods, salt, and alcohol. There was neither a specific calorie restriction nor a specific macronutrient ratio recommended (8). The recommendation outlined a minimum of 30 min of physical activity per day, alongside a reduction in sedentary behavior. For stress management, the recommendations included regular relaxation routines and taking breaks. Participants were further encouraged to form support groups, seek support from friends and family, and engage with a supportive community environment. A comprehensive description of the intervention and the recommendations has already been published (29).

The program encompassed 14 consecutive seminars, held twice weekly and each lasting 2 h, with all participants of the intervention group (IG). The seminars were conducted by the research group, and local health stakeholders from the health circle (e.g., general practitioners, representatives of the local sport club) were actively involved in the seminar units. The seminar sessions integrated a variety of BCTs to support participants' behavior change. The components of the intervention were retrospectively assigned to the behavior change technique taxonomy (v1) (30). Participants were informed about how their behaviors affect their health and emotions, aiming to raise awareness and foster motivation. Topics such as problem solving, habit formation, behavioral substitution, or distraction were components in the latter seminar units. In the seminars and during informal interactions, such as breaks, seminar facilitators employed verbal persuasion to stimulate participant's capability to change their health behavior and celebrate past success. They further encouraged strategies such as mental rehearsal and self-talk to promote SE. In addition, eight workshops with smaller groups (up to 20 participants each) were carried out (e.g., cooking courses, shopping tours, sports- and relaxation courses) to strengthen participants' practical skills. These workshops were implemented to shape knowledge through instruction, to give behavioral demonstrations, as well as to practice and rehearse new behaviors.

Each participant attended two individual coaching sessions, offered by health experts during health checks at baseline and after 10 weeks, to collaboratively set a concrete goal for the intervention period and plan its implementation using SMART-criteria. This goal was revisited and reviewed at the second coaching session to monitor progress and ensure alignment with participants' needs.

A healthy lifestyle handbook and a recipe booklet were provided to participants to reinforce and expand upon the content of the seminar units. As a prompt and cue, participants were given a laminated one-pager with the key lifestyle recommendations, where they could document their adherence to the recommendations on a daily basis. Social support formed another critical element of the intervention, offering participants both emotional and practical assistance. This included facilitation of peer-based initiatives, such as the formation of walking groups and the establishment of ongoing meetings of the participants beyond the intervention period.

The intensive phase was followed by a less-intensive alumni phase, which lasted 22 months. In this phase, participants attended monthly seminars. A newsletter was distributed to reinforce content from the intensive phase and support long-term health behavior change. During this phase, participants were encouraged to view themselves as role models within their environment and community.

### Control group

The CG received no intervention. For ethical reasons, the participants of the CG were informed about the results of their personal health check. The CG started 6 months later than the IG (start: April [IG] and October [CG] 2018) for organizational reasons, but the follow-up durations were equal in both groups.

### Ethical considerations

The study was registered in the German Clinical Trial Register (DRKS; reference: DRKS00018775; http://www.drks.de). It was performed in accordance with the Declaration of Helsinki, and the protocol was approved by the ethics committee of the Medical Association of Westphalia-Lippe and of the University of Muenster (Muenster, Germany; reference: 2018-171-f-S; approved 4 April 2018). Before being included in the study, the participants gave their written informed consent.

### Data collection

Data were collected at six measurement time points: baseline (*t*_0_), 10 weeks (*t*_1_; i.e., after the intensive phase of the HLCP-2 intervention), 6 months (*t*_2_), 12 months (*t*_3_), 18 months (*t*_4_), and 24 months (*t*_5_) (23). Participants completed questionnaires for socio-demographic and health-related parameters such as health behavior, health economic parameters, quality of life, physical activity, and wellbeing. Additionally, a semi-quantitative 3-day food record assessed the dietary intake of the participants. At each time point, blood parameters (e.g., cholesterol, triglycerides, and fasting glucose), anthropometric parameters (e.g., body weight, body mass index [BMI], height, and waist circumference) and vital signs (systolic and diastolic blood pressure) were recorded. Blood samples were obtained in a fasted state and analyzed at the University Hospital of Muenster. At the 24-months follow up (*t*_5_), the health check could not be conducted in person due to the COVID-19 pandemic and the contact restrictions in place at the time. Questionnaires were distributed by post. Therefore, only questionnaire data are available for this time point.

Self-reported health behaviors (eating and physical activity behaviors) were assessed retrospectively at each measurement time point using questionnaires. This analysis, however, focuses specifically on eating behavior. The questionnaires were based on the social-cognitive model of health action (Health Action Process Approach; HAPA) (31, 32) and validated in a pilot study (33).

Five HAPA constructs (action/coping planning, action SE, maintenance SE, recovery SE, outcome expectancy) were assessed. Table 1 presents an explanation of the constructs, exemplary items, the number of items per construct, minimum and maximum scores and Cronbach's alpha values. The response options were presented using four-point Likert scales. The scales for SE constructs ranged from “not confident (1)” to “completely confident (4)” and the scale for action/coping planning from “not at all (1)” to “firmly (4)”. Changes in health behavior were assessed by calculating sum scores based on multiple questions corresponding to the same HAPA construct. The internal consistency of the constructs was evaluated using Cronbach's alpha. Most constructs demonstrated high reliability, with Cronbach's alpha values >0.80, except for action self-efficacy at baseline (α = 0.723) and after 10 weeks (α = 0.785).

Eating behavior was assessed by means of semi-quantitative 3-day food records (two weekdays and 1 weekend day), which were based on portions of different food groups. The plant-based diet index (PDI) was calculated to assess adherence to dietary recommendations. Given the nature of our data, we assigned positive and negative scores based on food portions rather than using reverse scores based on quintiles, as used by Satija et al. (34). The PDI score was determined by subtracting the portions of all animal-based foods from the portions of all plant-based foods (34).


$$\begin{array}{r} PDI=number\;of\;animal\text{-}based\;food\;portions\;-\\ number\;of\;plant\text{-}based\;food\;portions \end{array}$$

Since plant-based foods are not inherently healthy, the healthy PDI (hPDI) and unhealthy PDI (uPDI) were also calculated [as previously reported: (23)]. In this paper, the hPDI was considered to evaluate healthy, plant-based dietary behavior. For calculating this score, the portions of animal-based and less healthy plant-based foods (e.g., sweetened beverages, sweets/desserts) were subtracted from portions of healthy plant-based foods (e.g., vegetables, fruits, nuts, whole grains, legumes). A higher score is considered favorable (34).


$$\begin{array}{r} hPDI=number\;of\;healthy\;plant\text{-}based\;food\;portions-\\ (number\;of\;less\;healthy\;plant\text{-}based\;food\;portions+\\ number\;of\;animal\text{-}based\;food\;portions) \end{array}$$

### Statistical analysis

The sample size calculation was based on the primary outcome parameter (change in body weight), that was already reported on in Koeder et al. (25). Missing data were not imputed and all available cases at each measurement time points were used. All tests were two-sided. While all results are described as statistically significant if the *p-*value is < 0.05, all analyses in the present paper should be considered exploratory. The *p-*values are thus interpreted as an indicator of the strength of evidence against the null hypothesis. For statistical analysis, groups were coded as 0 for the intervention group (IG) and 1 for the control group (CG).

Categorical data are given in absolute numbers and percentages (%), and quantitative parameters are reported in means ± standard deviation (SD). The Shapiro-Wilk test was used to test data for normality, with *p* < 0.05 describing a non-normal distribution. Baseline characteristics and the change of HAPA constructs throughout the study course were analyzed using appropriate statistical tests. Between-group differences were evaluated using the independent *t-*test for normally distributed continuous variables, whereas the Mann–Whitney U-test compared the between-group differences for non-normally distributed continuous variables. Fisher's exact test was performed for differences in categorical variables. Within-group changes were assessed using the paired *t-*test for normally distributed variables and the Wilcoxon-signed rank test for non-normally distributed variables. To account for multiple testing in inter- and intra-group comparisons, adjusted *p-*values using Holm–Bonferroni correction are reported.

For the covariate-adjusted comparison between the IG and CG, multiple linear regression (MLR) models were created taking the HAPA constructs as response variables. Potential confounders were identified based on their theoretical relevance (35, 36). The following confounders were used in the univariate analysis: sex, age, marital status, education level, blood parameters, vital signs, anthropometric parameters, smoking status, assignment to IG or CG, alcohol consumption, diagnosed disease, regular medication use, sleep quality, quality of life, perceived stress status, wellbeing, and HAPA constructs at baseline. A two-step modeling approach was used. First, univariate analyses with all confounders were performed as a screening step to identify candidate predictors for inclusion in the MLR. Second, forward selection with all significant confounders identified in the univariate analyses was performed. Final models were selected based on statistical significance (general linear *F-*test: *p* ≤ 0.05), the highest corrected *R*^2^ and the fewest covariates. The primary focus of the MLR models was to assess the effect of group allocation while adjusting for confounders. Residuals were visualized and checked for normality. To ensure the robustness of the observed group allocation effect, sensitivity analyses were conducted using a minimally adjusted model (covariates: group allocation, baseline values).

The Spearman correlation coefficient ρ was used to assess correlations between the change of HAPA constructs and hPDI. The guidelines from Cohen (37) were considered to assign the effect size (small: 0.2 < IρI ≤ 0.5, medium: 0.5 < IρI ≤ 0.8, large: 0.8 IρI ≤ 1.0). All analyses were performed in SPSS version 27 for Windows (SPSS Inc., Armonk, NY, USA).

## Results

**Figure 1:**
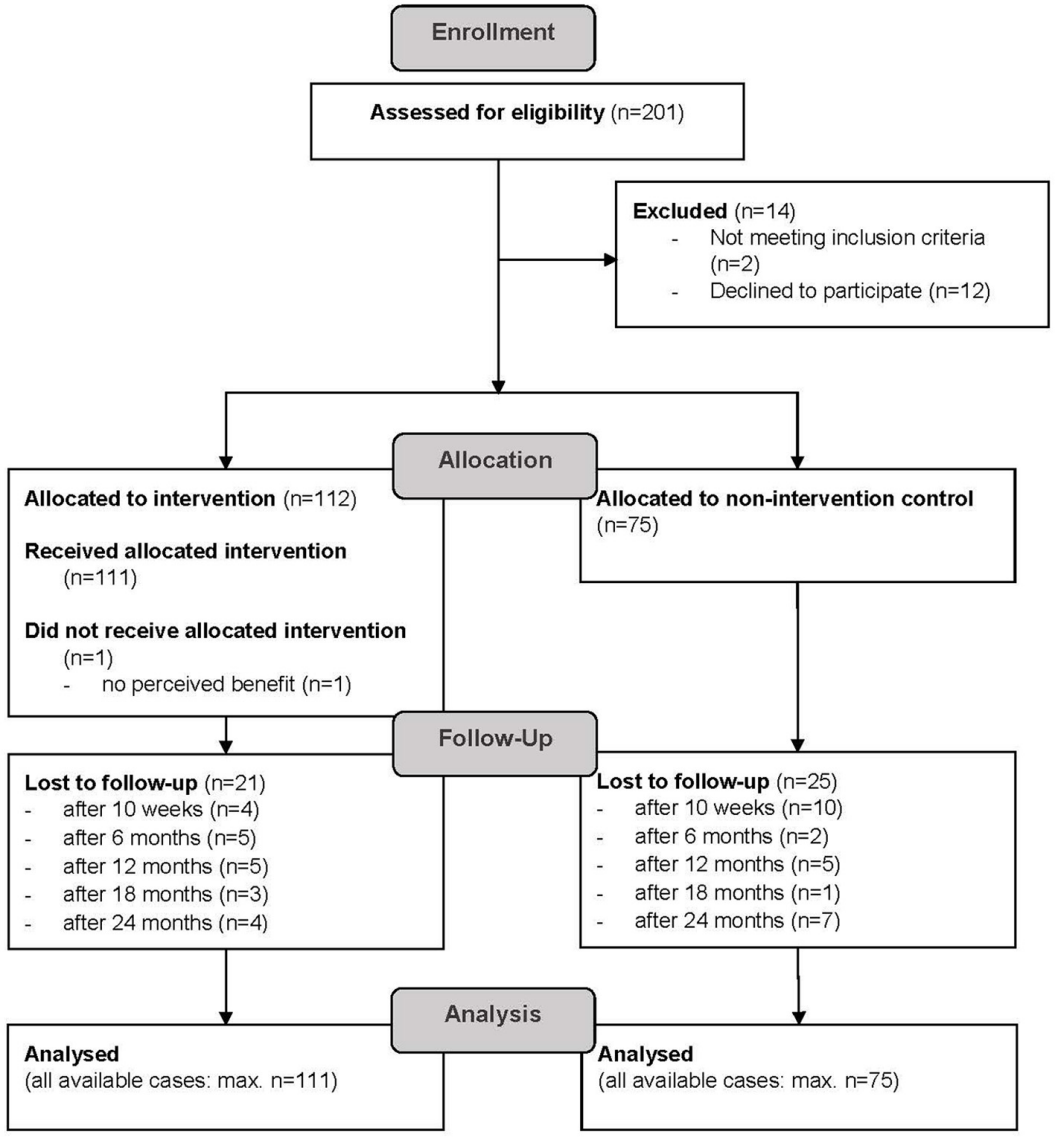
CONSORT participants' flow diagram; participants categorized as “lost to follow-up” did not show up to health checks or withdrew from the study.

Figure 1 shows the participants' flow through the study course. A total of 111 individuals participated in the IG, among whom 21 were lost to follow-up. The CG included 75 participants, 25 of whom were lost to follow-up.

### Baseline characteristics

Baseline characteristics of both study groups are presented in Table 2. Study participants were predominantly female (IG: 68.5%; CG: 60.0%), middle-aged (IG: 59.2 ± 8.9 years; CG: 54.0 ± 10.3 years), and overweight (mean BMI of 27.8 ± 5.3 [IG] and 28.6 ± 5.8 [CG]). The participants in the IG were older (*p* < 0.001) and had a higher standard of education (*p* = 0.001) compared to the CG. Anthropometric data, marital status, vital signs, blood parameters, and HAPA constructs were comparable between the study groups.

**Table 2:** Baseline characteristics (socioeconomic data, NCD risk profile and HAPA constructs) by study group.

**Variable**	**Intervention group**	**Control group**	***p*-value**
**Sociodemographic data**	*n = 111*	*n = 75*	
Age, years: mean ± SD	59.2 ± 8.9	54.0 ± 10.3	**< 0.001** ^**b**^
Female Sex: *n* (%)	76 (68.5)	45 (60.0)	0.273^a^
**Anthropometric data: mean** ±**SD**	*n = 110*	*n = 75*	
Body weight, kg: mean ± SD	82.0 ± 18.7	86.8 ± 19.6	0.064^c^
BMI, kg/m^2^: mean ± SD	27.8 ± 5.3	28.6 ± 5.8	0.424^c^
Waist circumference, cm: mean ± SD	99.1 ± 15.1	99.0 ± 16.9	0.942^b^
**Education level:** ***n*** **(%)**	*n = 108*	*n = 69*	**0.001** ^**a**^
Lower secondary school	20 (18.5)	26 (37.7)	
Secondary school	45 (41.7)	21 (30.4)	
University entrance qualification	22 (20.4)	19 (27.5)	
University degree	21 (19.4)	3 (4.3)	
**Marital status:** ***n*** **(%)**	*n = 108*	*n = 69*	0.539^a^
Married	87 (80.6)	60 (87.0)	
Partner, unmarried	6 (5.6)	3 (4.3)	
Single (not widowed)	11 (10.2)	3 (4.3)	
Single (widowed)	4 (3.7)	3 (4.3)	
**Vital signs: mean** ±**SD**	*n = 110*	*n = 75*	
Systolic BP, mm Hg	135.0 ± 15.6	131.3 ± 16.6	0.125^b^
Diastolic BP, mm Hg	81.7 ± 8.6	79.4 ± 9.8	0.140^c^
Pulse	68.7 ± 10.5	69.1 ± 10.1	0.757^b^
**Blood parameters: mean** ±**SD**	*n = 109*	*n = 75*	
TC, mg/dL	205.2 ± 37.5	205.9 ± 41.6	0.911^b^
HDL-C, mg/dL	64.3 ± 18.4	61.0 ± 18.0	0.200^c^
LDL-C, mg/dL	131.9 ± 34.7	136.9 ± 40.8	0.373^b^
TG, mg/dL	106.9 ± 53.8	119.8 ± 79.8	0.387^c^
Fasting glucose, mg/dL	100.0 ± 16.1	106.9 ± 30.0	0.472^c^
HbA1c, %	5.5 ± 0.5	5.6 ± 0.7	0.735^c^
**HAPA constructs**	*n = 107*	*n = 68*	
Action planning	16.0 ± 3.6	15.8 ± 3.5	0.590^c^
Action self-efficacy	11.9 ± 2.2	11.8 ± 2.4	0.839^c^
Maintenance self-efficacy	31.7 ± 6.2	31.4 ± 6.6	0.810^c^
Recovery self-efficacy	9.3 ± 1.8	9.3 ± 2.0	0.737^c^

SD, standard deviation; BMI, body mass index; BP, blood pressure; TC, total cholesterol; LDL-C, LDL cholesterol; HDL-C, HDL cholesterol; TG, triglycerides. ^a^Fisher**'**s exact test; ^b^Independent t-test; ^c^Mann–Whitney U-test. Bold values indicate statistical significance.

**Table 3:** Multiple linear regression models for action/coping planning (SE, standard error; ref., reference group).

**Dependent variable: change of action/coping planning**	**ß**	**SE**	***p*-value**
**10 weeks** ^a^			
Constant (_0_)	12.283	0.951	< 0.001
Group (ref. intervention)	-3.101	0.391	** < 0**.**001**
Action planning at baseline	-0.515	0.052	** < 0**.**001**
Triglycerides	-0.008	0.003	**0**.**005**
**6 months** ^b^			
Constant (_0_)	5.288	2.857	0.066
Group (ref. intervention)	-2.908	0.517	** < 0**.**001**
Action planning at baseline	-0.587	0.070	** < 0**.**001**
HbA1c, %	1.319	0.479	**0**.**007**
**12 months** ^c^			
Constant (_0_)	13.422	1.069	< 0.001
Group (ref. intervention)	-2.967	0.477	** < 0**.**001**
Action planning at baseline	-0.632	0.064	** < 0**.**001**
**18 months** ^d^			
Constant (_0_)	12.085	1.237	< 0.001
Group (ref. intervention)	-2.757	0.537	** < 0**.**001**
Action planning at baseline	-0.556	0.074	** < 0**.**001**
**24 months** ^e^			
Constant (_0_)	11.175	1.293	< 0.001
Group (ref. intervention)	-2.190	0.575	** < 0**.**001**
Action planning at baseline	-0.502	0.077	** < 0**.**001**

^a^Corrected R^2^ = 0.492; FS, general linear F-Test: *p* < 0.001; *n* = 160. ^b^Corrected R^2^ = 0.396; FS, general linear F-Test: *p* < 0.001; *n* = 155. ^c^Corrected R^2^ = 0.465; FS, general linear F-Test: *p* < 0.001; *n* = 148. ^d^Corrected R^2^ = 0.350; FS, general linear F-Test: *p* < 0.001; *n* = 141. ^e^Corrected R^2^ = 0.278; FS, general linear F-Test: *p* < 0.001; *n* = 136. All residuals are normally distributed. Bold values indicate statistical significance.

**Table 4:** Multiple linear regression models for action self-efficacy (SE, standard error; ref., reference group).

**Dependent variable: change in action self-efficacy**	**ß**	**SE**	***p*-value**
**10 weeks** ^a^			
Constant (_0_)	7.135	0.718	< 0.001
Group (ref. intervention)	-1.802	0.276	** < 0**.**001**
Action self-efficacy at baseline	-0.541	0.058	** < 0**.**001**
Sex (ref. male)	1.169	0.280	** < 0**.**001**
**6 months** ^b^			
Constant (_0_)	4.038	1.885	0.034
Group (ref. intervention)	-1.117	0.331	**0**.**001**
Action self-efficacy at baseline	-0.536	0.070	** < 0**.**001**
Triglycerides	-0.007	0.003	**0**.**010**
HbA1c, %	0.780	0.308	**0**.**012**
**12 months** ^c^			
Constant (_0_)	6.836	0.899	< 0.001
Group (ref. intervention)	-1.252	0.345	** < 0**.**001**
Action self-efficacy at baseline	-0.463	0.074	** < 0**.**001**
**18 months** ^d^			
Constant (_0_)	7.970	1.335	< 0.001
Croup (ref. intervention)	-1.321	0.339	** < 0**.**001**
Action self-efficacy at baseline	-0.532	0.073	** < 0**.**001**
Weight	-0.024	0.009	**0**.**008**
Quality of sleep	0.484	0.206	**0**.**020**
**24 months** ^e^			
Constant (_0_)	5.541	1.142	< 0.001
Group (ref. intervention)	-0.996	0.358	**0**.**006**
Action self-efficacy at baseline	-0.484	0.081	** < 0**.**001**
HDL-cholesterol	0.022	0.010	**0**.**028**

^a^Corrected R^2^ = 0.450; FS, general linear F-Test: *p* < 0.001; *n* = 166. ^b^Corrected R^2^ = 0.335; FS, general linear F-Test: *p* < 0.001; *n* = 157. ^c^Corrected R^2^ = 0.253; FS, general linear F-Test: *p* < 0.001; *n* = 149. ^d^Corrected R^2^ = 0.345; FS, general linear F-Test: *p* < 0.001; *n* = 143. ^e^Corrected R^2^ = 0.256; FS, general linear F-Test: *p* < 0.001; *n* = 137. All residuals are normally distributed; HDL, high density lipoprotein. Bold values indicate statistical significance.

**Table 5:** Multiple linear regression models for maintenance self-efficacy (SE, standard error; ref., reference group).

**Dependent variable: change of maintenance self-efficacy**	**ß**	**SE**	***p*-value**
**10 weeks** ^a^			
Constant (_0_)	16.309	2.119	< 0.001
Group (ref. intervention)	-4.367	0.871	** < 0**.**001**
Maintenance self-efficacy at baseline	-0.381	0.065	** < 0**.**001**
**6 months** ^b^			
Constant (_0_)	3.940	5.081	0.439
Group (ref. intervention)	-2.188	0.881	**0**.**014**
Maintenance self-efficacy at baseline	-0.419	0.066	** < 0**.**001**
HbA1c, %	2.347	0.817	**0**.**005**
**12 months** ^c^			
Constant (_0_)	16.588	2.247	< 0.001
Group (ref. intervention)	-2.145	0.930	**0**.**022**
Maintenance self-efficacy at baseline	-0.405	0.068	** < 0**.**001**
**18 months** ^d^			
Constant (_0_)	15.998	2.615	< 0.001
Group (ref. intervention)	-2.110	1.050	**0**.**046**
Maintenance self-efficacy at baseline	-0.369	0.079	** < 0**.**001**
**24 months** ^e^			
Constant (_0_)	10.720	3.213	0.001
Group (ref. intervention)	-1.726	1.086	0.115
Maintenance self-efficacy at baseline	-0.388	0.086	** < 0**.**001**
HDL-cholesterol	0.084	0.030	**0**.**006**

^a^Corrected R^2^ = 0.261. FS, general linear F-Test: *p* < 0.001; *n* = 161. ^b^Corrected R^2^ = 0.265. FS, general linear F-Test: *p* < 0.001; *n* = 152. ^c^Corrected R^2^ = 0.207. FS, general linear F-Test: *p* < 0.001; *n* = 147. ^d^Corrected R^2^ = 0.158. FS, general linear F-Test: *p* < 0.001; *n* = 141. ^e^Corrected R^2^ = 0.161. FS, general linear F-Test: *p* < 0.001; *n* = 135. All residuals are normally distributed; HDL, high density lipoprotein. Bold values indicate statistical significance.

**Table 6:** Multiple linear regression models for recovery self-efficacy (SE, standard error; ref., reference group).

**Dependent variable: change of recovery self-efficacy**	**ß**	**SE**	***p*-value**
**10 weeks** ^a^			
Constant (_0_)	4.730	0.832	< 0.001
Group (ref. intervention)	-1.104	0.240	** < 0**.**001**
Recovery self-efficacy at baseline	-0.556	0.061	** < 0**.**001**
Total cholesterol	0.008	0.003	**0**.**011**
**6 months** ^b^			
Constant (_0_)	2.053	1.511	0.176
Group (ref. intervention)	-0.919	0.266	**0**.**001**
Recovery self-efficacy at baseline	-0.508	0.069	** < 0**.**001**
HbA1c, %	0.639	0.248	**0**.**011**
**12 months** ^c^			
Constant (_0_)	5.494	0.776	< 0.001
Group (ref. intervention)	-0.456	0.318	0.153
Recovery self-efficacy at baseline	-0.505	0.080	** < 0**.**001**
Smoking status	-0.481	0.214	**0**.**026**
**18 months** ^d^			
Constant (_0_)	5.176	0.822	< 0.001
Group (ref. intervention)	-0.821	0.320	**0**.**011**
Recovery self-efficacy at baseline	-0.437	0.082	** < 0**.**001**
Regular medication intake	-0.676	0.322	**0**.**038**
**24 months** ^e^			
Constant (_0_)	4.655	0.849	< 0.001
Group (ref. intervention)	-0.626	0.340	0.068
Recovery self-efficacy at baseline	-0.439	0.088	** < 0**.**001**

^a^Corrected R^2^ = 0.400. FS, general linear F-Test: *p* < 0.001; *n* = 165. ^b^Corrected R^2^ = 0.312. FS, general linear F-Test: *p* < 0.001; *n* = 157. ^c^Corrected R^2^ = 0.235. FS, general linear F-Test: *p* < 0.001; *n* = 149. ^d^Corrected R^2^ = 0.197. FS, general linear F-Test: *p* < 0.001; *n* = 143. ^e^Corrected R^2^ = 0.160. FS, general linear F-Test: *p* < 0.001; *n* = 138. All residuals are normally distributed. Bold values indicate statistical significance.

Overall, a high level of seminar attendance by participants in the IG was observed. Eighty eight percent of the study participants (*n* = 98) attended at least half of the seminars and 59% (*n* = 65) participated in at least 11 of the 14 seminars.

### HAPA constructs of behavior change

**Figure 2:**
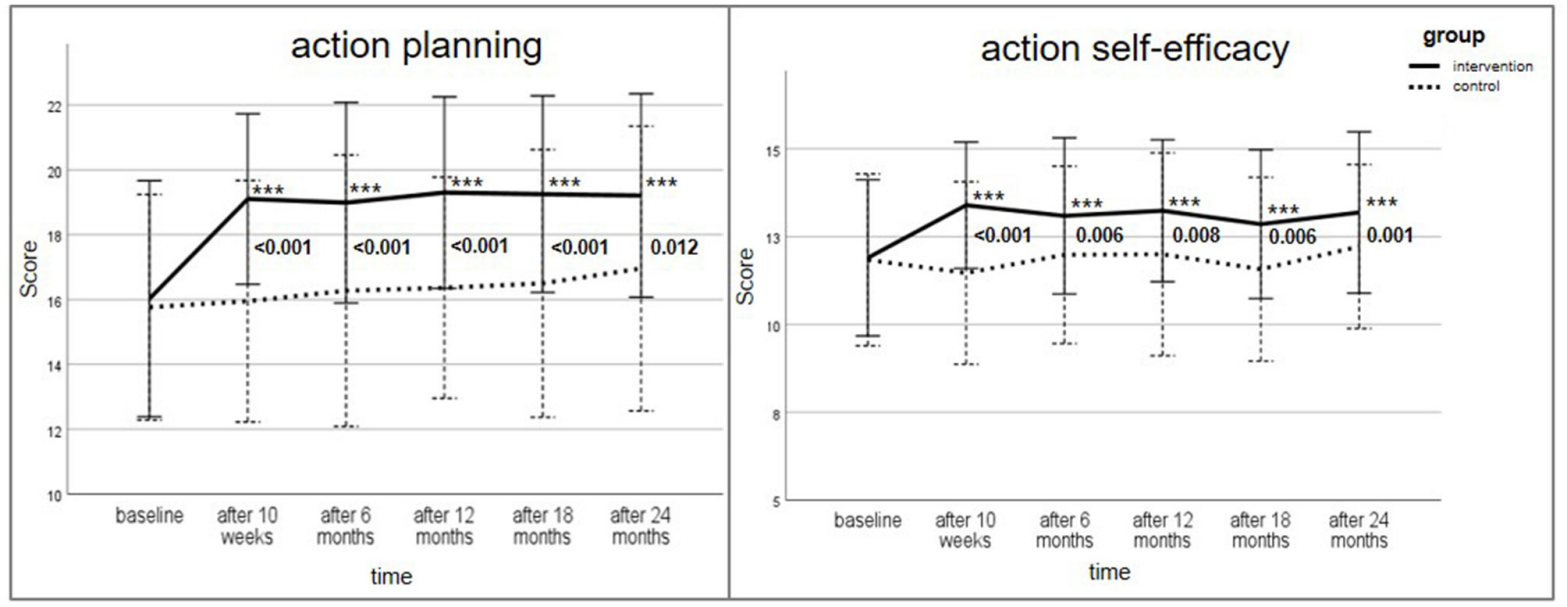
Change of action/coping planning score (max. score 24) and action self-efficacy (max. score 16) for intervention and control group over the study period; Data are presented as mean ± standard deviation (SD), with error bars representing SD; All *p-*values were adjusted using Holm–Bonferroni correction for multiple testing; Wilcoxon-test with *p ≤ 0.05, **p ≤ 0.01, ***p ≤ 0.001 for within group comparison to baseline; Mann–Whitney-U-Test for between-group comparison of change of score (reference: baseline) provided in numbers. Bold values indicate statistical significance.

**Figure 3:**
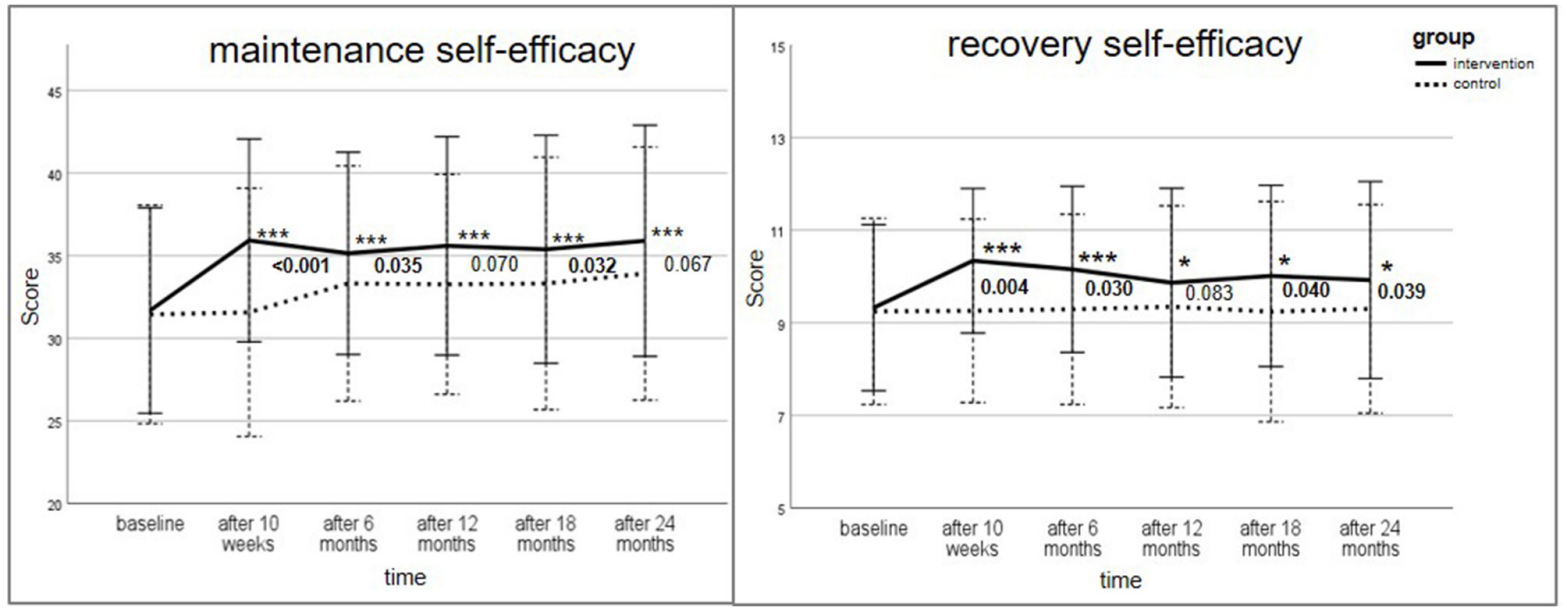
Change of maintenance self-efficacy score (max. score 48) and recovery self-efficacy (max score 12) for intervention and control group over the study period; Data are presented as mean ± standard deviation (SD), with error bars representing SD; All *p-*values were adjusted using Holm–Bonferroni correction for multiple testing; Wilcoxon-test for within-group differences with *p ≤ 0.05, **p ≤ 0.01, ***p ≤ 0.001 for comparison to baseline; Man–Whitney U-Test for between-group comparison of change of score (reference: baseline) provided in numbers. Bold values indicate statistical significance.

The psychological constructs of the HAPA model (action/coping planning, action SE, maintenance SE, recovery SE) are shown in Figures 2 and 3. Supplementary material 1 provides a detailed tabular presentation of the results. To adjust for multiple comparisons, *p-*values were corrected using the Holm–Bonferroni method.

At baseline, the score of action/coping planning (max. score: 24) was comparable in both groups (IG: 16.0 ± 3.6; CG: 15.8 ± 3.5; *p* = 0.589). In the IG, the score increased considerably to 19.1 ± 2.6 after the intensive phase of the lifestyle intervention (*p* ≤ 0.001) and remained significantly higher over the study period. In the CG, however, the score did not noticeably change.

The HAPA constructs action SE (max. score: 16), maintenance SE (max. score: 48) and recovery SE (max. score: 12) were likewise analyzed. The scores increased significantly in the IG at all measurement time points compared to baseline (*p* ≤ 0.05) with the highest change after the intensive lifestyle intervention. All intra-group comparisons in the CG revealed no statistical significance.

Between-group differences regarding the change of action/coping planning (from baseline) were significant at all time points (*p* ≤ 0.001; after 24 months: *p* = 0.012). The between-group comparison for action SE reached statistical significance over the whole study period (*t*_1_: *p* ≤ 0.001; *t*_2_: *p* = 0.006; *t*_3_: *p* = 0.008; *t*_4_: *p* = 0.006; *t*_5_: *p* = 0.001). Maintenance SE was significant between the groups after 10 weeks (*p* < 0.001), 6 months (*p* = 0.035) and 18 months (*p* = 0.032). The change of recovery SE score (reference: baseline) between the IG and CG was significantly different, with one exception after 12 months (*p* = 0.083).

### Multiple linear regression models

To adjust for confounders, MLR models were estimated, taking the four HAPA constructs as dependent variables (see Tables 3-6). Action/coping planning, action SE and maintenance SE differed significantly between the groups in all final models, with three exceptions (maintenance SE after 24 months; recovery SE after 12 and 24 months). Affiliation in the CG was associated with a smaller increase in HAPA constructs over the study period compared to IG. For example, the action/coping planning score was on average 3.101 points lower in the CG than in the IG after 10 weeks. The betas for action/coping planning almost gradually, but only slightly decreased in the follow-up measurements (β = −2.190 at 24 months, *p* < 0.001). The betas for action SE were likewise decreasing (not linear) through the study course (*t*_0_: β = −1.802, *p* < 0.001; *t*_5_: β = −0.996, *p* = 0.006). At baseline, maintenance SE score was on average 4.367 points lower in the CG than in the IG (*p* < 0.001). Betas for recovery SE were comparably small (*t*_0_: β = −1.104, *p* < 0.001). Additionally, a lower score at baseline (e.g., a less well-developed SE or action/coping planning) was a predictor for a greater increase in the corresponding score for all HAPA constructs at all measurement time points (*p* < 0.001). Most of the covariates, i.e., vital parameters, waist circumference, education level, marital status or alcohol consumption, had a significant influence on the HAPA constructs. The results remained robust when tested through sensitivity analyses, including a minimally adjusted model.

### Correlations between HAPA constructs and eating behavior

Bivariate correlations between the change of the score of the HAPA constructs and the hPDI as a parameter for the actual intake of healthy, plant-based foods are presented in Table 7. Only weak to moderate correlations were observed. After 10 weeks, in the IG, the association between the change of hPDI and recovery SE was significant (*p* = 0.004) with a moderate positive correlation, i.e., a greater change of hPDI was correlated with a greater change of recovery SE. However, after 24 months, the correlation of hPDI with action/coping planning (*p* = 0.006), action SE (*p* = 0.014), and recovery SE (*p* = 0.023) was significant. In the CG, action SE and hPDI correlated significantly after 10 weeks (*p* = 0.034). After 24 months, a negative correlation of hPDI with maintenance SE (*p* = 0.025) was observed, i.e., a greater change of hPDI was correlated with a smaller change of maintenance SE.

**Table 7:** Bivariate correlations between the change of HAPA constructs and the change of healthy plant-based diet index (hPDI).

	**Change of hPDI**							
	**After 10 weeks** ^a^				**After 24 months** ^a^			
	**IG**		**CG**		**IG**		**CG**	
**Change of**	ρ	***p*** **-value**	ρ	***p*** **-value**	ρ	***p*** **-value**	ρ	***p*** **-value**
Action/coping planning^b^	0.090	0.379	0.071	0.610	0.309	**0**.**006**	−0.181	0.239
Action SE^b^	0.177	0.078	0.284	**0**.**034**	0.272	**0**.**014**	0.106	0.494
Maintenance SE^b^	0.121	0.239	0.129	0.348	0.174	0.123	−0.337	**0**.**025**
Recovery SE^b^	0.289	**0**.**004**	−0.180	0.185	0.255	**0**.**023**	−0.179	0.245

^a^Compared to baseline. ^b^Change after 10 weeks/24 months compared to baseline. hPDI, healthy plant-based diet index; SE, self-efficacy; IG, intervention group; CG, control group; ρ, Spearman correlation coefficient (Spearman's rho). Bold values indicate statistical significance.

### Outcome expectancy

Participants were asked about the advantages and disadvantages of implementing a healthy plant-based diet. At baseline, participants indicated that a change in dietary behavior was beneficial (almost right or exactly right) for their blood parameters (IG: 97.2%; CG: 94.2%), blood pressure (IG: 91.6%; CG: 84.1%) and weight (IG: 97.2%; CG: 97.1%). In both groups, the most prevalent negative outcome expectancies were “It is stressful to buy the right products for a healthy diet” (IG: 62.0%; CG: 46.4%) and “It is very complex for me to eat healthy” (IG: 32.4%; CG: 23.6%). However, these parameters decreased in the IG after the intensive intervention phase (stressful: 38.7%; complex: 23.6%). Less common answers from the participants at baseline were “a healthy diet causes a loss of quality of life” (IG: 15.8%, CG: 14.4%), “…burdens me financially” (IG: 12.1%; CG: 22.4%) or “…interferes with my social life” (IG: 2.7%, CG: 5.4%).

## Discussion

This study investigated the influence of the HLCP-2 intervention on health behavior, particularly with regard to a healthy diet. In almost all HAPA constructs assessed, the scores increased significantly in the IG compared to baseline and control, with the highest increase after the intensive phase of 10 weeks. The results remained significant even with adjustments for covariates through the MLR for almost all measurement time points.

### HAPA stages of behavior change

Particularly notable in this study were the consistently strong effects of the intervention on the constructs of action/coping planning and action SE. Across all measurement time points, these constructs showed significant improvements in the IG compared to both baseline and the CG. The sustained and robust effects observed for action/coping planning underline the central role of this construct as a predictor of health behavior change, as it facilitates the translation of intentions into concrete behavioral strategies (4, 18, 38). Similarly, the intervention led to consistently high increases in action SE, emphasizing its importance in developing a strong intention to change behavior (15). The findings regarding maintenance SE were less consistent compared to other constructs, particularly over the longer term. While the IG showed significant improvements in maintenance SE at some time points, these effects were not consistently observed across the entire study duration. One potential explanation for this observation is the slight, though non-significant, increase in maintenance SE within the control group, which may have reduced the relative difference between groups. Allocation to the intervention group was not an independent predictor for change of recovery SE after 12 and 24 months. The long-term effects for recovery SE can be described as relatively modest, which could be related to the fact that recovery SE is most functional in phases in which a failure or a setback takes place (14, 39).

Miller et al. (20) analyzed the effects of a Worksite Diabetes Prevention Intervention on health behavior using social cognitive variables. Action planning, action and maintenance (coping) SE improved significantly after the intervention (*p* < 0.05), but not at the 3-months follow-up. Regarding intensity and topics of lifestyle modifications, the two interventions are comparable. The consideration of the importance of community support, alumni-meetings, and newsletters, as well as the longer follow-up duration of the HLCP-2 intervention, may have led to the more lasting effect of the HLCP intervention on the HAPA constructs compared to the Worksite Diabetes Prevention Program.

An explanation why the change of HAPA stages was successful could be that health behaviors that are experienced as pleasant are more likely to be repeated (upward spiral theory of lifestyle changes) (40). The HLCP-2 intervention was designed to be practically oriented, through workshops such as cooking courses or supermarket trips, and engaging, e.g., via physical activity sessions with community-building games like rope pulling, to enhance participants' enjoyment of a health-promoting lifestyle.

The order of the analyzed constructs reflects their theorized importance and order within the HAPA framework for initiating and maintaining behavior change. Action/coping planning and action SE were emphasized due to their relevance in bridging intentions to initial action during the early stages of the intervention, while maintenance SE and recovery SE became more important for explaining long-term behavioral adherence. Outcome expectancy, reported descriptively in this study, provided additional context for understanding how participants evaluated the advantages and disadvantages for implementing a healthy plant-based diet but was not a primary focus of the analysis.

### Correlations between HAPA constructs and eating behavior

We further assessed, whether the change of behavior stages is reflected in the actual eating behaviors of the participants. As previously published, the HLCP-2 intervention significantly improved the hPDI in the IG after 10 weeks and 12 months (23). The bivariate correlations between the change of the HAPA constructs and the change of hPDI showed that the improvements of the hPDI are partly attributable to improvements in action/coping planning or action and recovery SE. It is interesting that this correlation was especially visible after 24 months. However, there were small correlations for all HAPA constructs. This supports the hypotheses that behaviors such as food choices are highly complex, have many contributing factors and that behavior change is a long-term process, which is difficult to monitor in research. Furthermore, long-term behavior change should be assessed, especially after intensive lifestyle interventions.

### Influence of different BCTs on behavior change

It is beyond the scope of this analysis to determine which specific BCT used in the intervention contributed to the observed increase in the HAPA constructs. It is only possible to hypothesize about potential mechanisms linking the BCTs employed in this study to the observed effects on the HAPA constructs. Recent research has identified several BCTs, as outlined in the Behavior Change Technique Taxonomy (v1), that are effective in enhancing SE. These are graded tasks, verbal persuasion about capability, focus on past success, demonstration of behavior, problem solving, behavioral practice/rehearsal and reduction of negative emotions (13). In this study, we incorporated nearly all of the BCTs that have been shown to enhance SE, with the exception of graded tasks. In the seminar units, verbal persuasion about capability, focus on past success and problem-solving were used. Individual hurdles and barriers, as well as possible solution approaches, were addressed, which may have strengthened participants' maintenance and recovery SE. Additionally, the practical workshops were a central component of the intervention and included BCTs such as demonstration of behaviors and behavioral practice/rehearsal.

In this study, a variety of BCTs were incorporated. In terms of increasing physical activity, interventions with a larger number of BCTs used seemed to be more effective (41). However, whether these findings can be applied to dietary behavior remains an open question requiring further research.

The impact of different BCTs on resources/behavior change mechanisms and actual behavior warrants further investigation. However, disentangling these correlations remains methodologically challenging. Approaches such as systematic reviews, meta-analyses, or qualitative research could provide valuable insights into the underlying mechanisms of behavior change.

### Implications and future research

Existing models of health behavior change, like the HAPA model, often focus on linear stages that involve cognitive engagement. However, behavior change is often based on automatic, unconscious or affective processes. One model that focuses on patients' resources and considers the different processes is the Behavior Change Resource Model (BCRM) (12, 42), which could be used in further investigations of health behavior change in lifestyle interventions.

An even longer observation of the participants would be beneficial for evaluating the health-related behavior without the impact of the HLCP-2 intervention, since the alumni phase included monthly seminars and newsletters. With this longer follow-up duration, the extent of which the participants implemented a healthy diet in their lives after the intervention could be evaluated. For this reason, a further follow-up assessment of the study cohort is planned. Analysis of data on behavioral stages and SE in terms of physical activity was conducted and will be published separately.

### Strengths and limitations

The main strengths of the present study are the non-intervention CG and the complex real-world setting. These settings (also called real-world laboratories) can help transfer scientific knowledge into practice by carrying out interventions in a real-life context (e.g., in a community setting) rather than in a clinical or lab environment (43). A further strength of the study is the long observation period of participants (2 years), as many studies tend to only monitor participants during or right after the intensive intervention. This longer time span allowed us to evaluate the dynamic role of the HAPA constructs, especially maintenance and recovery SE, over time. In addition, the use of two-sided tests for hypothesis testing reflects a conservative and robust approach. While the intervention was expected to produce effects in one direction (improvement of HAPA scores), this choice allowed us to detect unexpected or counterintuitive findings across the entire 2-year period. This approach ensured a more comprehensive interpretation of the results, particularly in a real-world setting where behavior change is inherently complex.

The study has several limitations.

#### Study design

The lack of randomization [as described previously: (25)] is one of the main limitations of the study. Although we adjusted for potential confounders in the MLR, selection bias may have influenced the results. Due to the voluntary nature of participation in the study, it can be assumed that individuals who chose to participate may be more health-conscious, motivated to change their behavior or already more engaged in a healthy lifestyle compared to the general population or a high-risk target group in a clinical setting. As a consequence, intervention effects may be overestimated. A further limitation is that the CG started 6 months after the IG, whereby the interval between the measurement time points was identical. This timing discrepancy may have introduced bias due to seasonal variations in the targeted behavior and associated health markers, which tend to improve in summer months (44). However, the present study did not reveal any consistent seasonal trends in the risk parameters (data not shown).

#### Self-reporting

Social desirability may have played a role in answering the questions regarding health behavior, as the change of the lifestyle was a (mediated) goal of the intervention. Participants may have overreported HAPA constructs, potentially leading to overestimation of intervention effectiveness. Self-administered and anonymized questionnaires were used in the study to minimize the influence of social desirability on the participants' responses by reducing concerns about judgment or negative consequences (45). The assessment of food intake in a 3-day food record has a risk of self-reporting bias by underreporting unhealthy and overreporting healthy foods. To address this concern, the food score hPDI was used rather than individual food groups.

#### Handling of missing data

The handling of missing data represents a potential limitation in this study, particularly given the loss to follow-up of 21 out of 111 participants in the intervention group and 25 out of 75 participants in the control group. However, analyses of missing data patterns using Little's MCAR test indicated that the data were missing completely at random (*p* = 0.97). This finding suggests that the missingness was distributed randomly across the dataset and, therefore, unlikely to introduce systematic bias into the results. To further mitigate bias, an “all available cases” analysis was used to include as much data as possible while avoiding the potential selection bias that can occur with complete-case analysis. Additionally, this approach avoided introducing potentially speculative estimates or assumptions that could have influenced the validity of the findings.

#### Recruitment

In addition, the study groups were different in terms of age and educational level at baseline. A higher education level of the IG could have had an impact on the health and health behavior of the participants (46) and led to a better implementation of the lifestyle recommendations in everyday life.

#### COVID-19 pandemic

Another limitation of the study is the occurrence of the COVID-19 pandemic, as scheduled health checks could not take place due to contact restrictions [as described previously: (23)]. The findings of a systematic review indicated that the COVID-19 pandemic had a significant impact on dietary habits. A notable increase in snack frequency has been observed, which was characterized by a preference for sweet and ultra-processed foods, and a decline in the consumption of fruits, vegetables and other fresh foods (47). The impact of the pandemic on HAPA constructs regarding dietary behavior remains to be explored. The results of the present study suggest that, in this particular sub-group, the pandemic did not exert a significant influence on the HAPA constructs.

## Conclusion

Our study investigated whether the HLCP-2 intervention was able to change citizens' eating behavior, as assessed by variables derived from the HAPA model, in a community-based lifestyle intervention. The HLCP-2 was able to increase action/coping planning as well as action, maintenance, and recovery SE. However, the correlation between the HAPA constructs and the change in food choices toward healthy, plant-based foods was only moderate. Future studies should investigate which factors influence actual healthy food choices in the everyday life of study participants and how such behavior can be implemented in the long term. Lifestyle interventions should be developed on the basis of these results to achieve the greatest possible health improvement for participants.

## Data Availability

The raw data supporting the conclusions of this article will be made available by the authors, without undue reservation.
