# Supplementary material for: Effect of a community-based lifestyle intervention on predictors of behavior change regarding a healthy plant-based diet—The Healthy Lifestyle Community Program (cohort 2)
Source: Front Public Health. 2025 Oct 6;13:1560376. doi: 10.3389/fpubh.2025.1560376 (PMC12535869; doi:10.3389/fpubh.2025.1560376)
Supplement: Supplementary file 1 [file Table_1.docx]

| All p-values |  | Baseline | 10 weeks | p-value^#^ | 6 months | p-value^#^ | 12 months | p-value^#^ | 18 months | p-value^#^ | 24 months | p-value^#^ |
| --- | --- | --- | --- | --- | --- | --- | --- | --- | --- | --- | --- | --- |
| Action planning (24) | **IG** | 16.0 ± 3.6 (n = 107) | **19.1 ± 2.6***** (n = 105) | **< 0.001** | **19.0 ± 3.1***** (n = 98) | **< 0.001** | **19.3 ± 3.0***** (n = 96) | **< 0.001** | **19.3 ± 3.0***** (n = 91) | **< 0.001** | **19.2 ± 3.1***** (n = 90) | **0.012** |
|  | **CG** | 15.8 ± 3.5 (n = 68) | 16.0 ± 3.7 (n = 60) |  | 16.3 ± 4.2 (n = 62) |  | 16.4 ± 3.4 (n = 55) |  | 16.5 ± 4.1 (n = 54) |  | 17.0 ± 4.4 (n = 50) |  |
| Action  self-efficacy  (16) | **IG** | 11.9 ± 2.2 (n = 108) | **13.4 ± 1.8*****  (n = 106) | **< 0.001** | **13.1 ± 2.2***** (n = 98) | **0.006** | **13.2 ± 2.0***** (n =96) | **0.008** | **12.9 ± 2.1***** (n = 91) | **0.006** | **13.2 ± 2.3***** (n = 90) | **0.001** |
|  | **CG** | 11.8 ± 2.4 (n = 69) | 11.5 ± 2.6 (n = 62) |  | 12.0 ± 2.5 (n = 62) |  | 12.0 ± 2.9 (n = 55) |  | 11.6 ± 2.6 (n = 54) |  | 12.2 ± 2.3 (n = 50) |  |
| Maintenance self-efficacy (48) | **IG** | 31.7 ± 6.2 (n = 107) | **35.9 ± 6.1***** (n = 104) | **< 0.001** | **35.1 ± 6.1***** (n = 97) | **0.035** | **35.6 ± 6.6*****  (n = 96) | 0.070 | **35.4 ± 6.9***** (n = 91) | **0.032** | **35.9 ± 7.0***** (n = 90) | 0.067 |
|  | **CG** | 31.4 ± 6.6 (n = 68) | 31.6 ± 7.5 (n = 60) |  | 33.3 ± 7.1 (n = 60) |  | 33.3 ± 6.7 (n = 53) |  | 33.3 ± 7.6 (n = 54) |  | 33.9 ± 7.7 (n = 50) |  |
| Recovery self-efficacy (12) | **IG** | 9.3 ± 1.8 (n = 108) | **10.3 ± 1.6***** (n = 106) | **0.004** | **10.2 ± 1.8***** (n = 98) | **0.030** | **9.9 ± 2.0*** (n = 96) | 0.083 | **10.0 ± 2.0*** (n = 91) | **0.040** | **9.9 ± 2.1*** (n = 90) | **0.039** |
|  | **CG** | 9.3 ± 2.0 (n = 69) | 9.3 ± 2.0 (n = 62) |  | 9.3 ± 2.1 (n = 62) |  | 9.4 ± 2.2 (n = 55) |  | 9.2 ± 2.4 (n = 54) |  | 9.3 ± 2.3 (n = 50) |  |
| All p-values were adjusted using Holm-Bonferroni correction for multiple testing  Wilcoxon-test for within-group differences with *p<0.05. **p<0.01. ***p<0.001 for within group comparison to baseline; Mann-Whitney-U test for between-group differences; # p-value for between-group comparison of the scores (reference: t_0_); IG: intervention group; CG: control group; SD: standard derivation | | | | | | | | | | | | |

**Appendix 1:** Psychological constructs (max. score) over the study period (baseline, 10 weeks, 6, 12, 18 and 24 months) in the IG and CG
